# Modeling of Hyper-Viscoelastic Properties of High-Damping Rubber Materials during the Cyclic Tension and Compression Process in the Vertical Direction

**DOI:** 10.3390/polym14245395

**Published:** 2022-12-09

**Authors:** Bowen Chen, Junwu Dai, Zhipeng Shao

**Affiliations:** 1 Key Laboratory of Earthquake Engineering and Engineering Vibration, Institute of Engineering Mechanics, China Earthquake Administration, Harbin 150086, China; 2 Key Laboratory of Earthquake Disaster Mitigation, Ministry of Emergency Management, Harbin 150086, China

**Keywords:** urban rail transit system, high-damping rubber isolation bearing, high-damping rubber material, isolation performance in the vertical direction, hyper-viscoelastic properties

## Abstract

With the rapid development of the economy and urbanization, the construction of the urban rail transit system has had a great impact on the work, life, and health of residents in buildings along the rail transit line. Thus, it is particularly urgent and necessary to develop base isolation technologies to control and reduce the impact of vibrations of rail transit systems on building structures. High-damping rubber isolation bearings have shown significant effectiveness in the reduction of this impact, and their isolation performance mainly depends on the mechanical and damping energy dissipation characteristics of the high-damping rubber material. This paper aims to investigate the hyper-viscoelastic properties of the high-damping rubber material used for high-damping rubber isolation bearings during the cyclic tension and compression process in the vertical direction. These properties include hyperelastic parameters, viscoelastic coefficients, and the relaxation times of the material. For this purpose, uniaxial cyclic tension and compression tests were conducted. A three-element Maxwell rheological model combining a strain energy density function was proposed for modeling the hyper-viscoelastic behaviors of the materials during the cyclic tension and compression process. Based on the obtained results, an iterative identification procedure was used to determine the constitutive parameters of the material for each loading-unloading cycle. The aforementioned parameters were further expressed as a function of the number of cycles. New insights into hyper-viscoelastic property changes in this high-damping rubber material during the cyclic tension and compression process were gained in this work. These investigations could facilitate the development of computational tools, which would regulate fundamental guidelines for the better controlling and optimization of the isolation performance of the high-damping rubber material used for high-damping rubber isolation bearings, which have a wider perspective of applications in the urban rail transit system.

## 1. Introduction

With the rapid development of the economy and urbanization, the construction of the urban rail transit system has become one of the main sources of urban noise and vibration pollution [1]. Building structures are frequently built in inner-city areas close to subways or other transportation facilities. The noise and vibrations generated by the rail transit system are transmitted to the adjacent building structures through the ground and the building foundation, thereby greatly affecting the work, life, and health of residents in buildings along the rail transit line [2]. Therefore, to effectively control and reduce the response of building structures to the vibrations of the rail transit system, it is particularly urgent and necessary to develop base isolation technologies of excellent isolation performance. To this end, considerable efforts have been invested in the research and development of this technology. Different types of isolation devices have been invented, among which rubber isolation bearings have shown great effectiveness in the reduction of the response of building structures to the vibrations of the rail transit system [3]. A three-dimensional isolator, which was composed of connecting plates, vertical rubber pads, and lead-core rubber isolation bearings, was developed. This isolation device could reduce the response of the superstructure of residential buildings to subway vibrations, which are built above the subway platform [2]. It showed that the maximum acceleration response of the superstructure of buildings installed with this isolator was ten times lower than that of the subway platform, and the vibration was attenuated to a maximum of 25 decibels. The effectiveness of laminated rubber isolation bearings in the vibration isolation of building structures was investigated using scale model tests [4]. By reducing and stabilizing the acceleration transfer coefficient of the vibration, it showed that high-damping rubber isolation bearings could effectively reduce the subway vibration. Furthermore, the subway vibration transmitted into building structures is mainly attributed to its vertical component [5]; hence, the isolators should have the sufficient compressive bearing capacity and a small vertical stiffness [6]. A correlation of the vertical stiffness and the damping characteristics with design variables, such as surface pressure, loading frequency, and temperature, under the subway vibration was studied on four types of rubber isolation bearings [3]. Through testing the vertical isolation performance, it was found that the mechanical properties of high-damping rubber isolation bearings greatly change with the surface pressure, loading frequency, and temperature.

**Figure 1:**
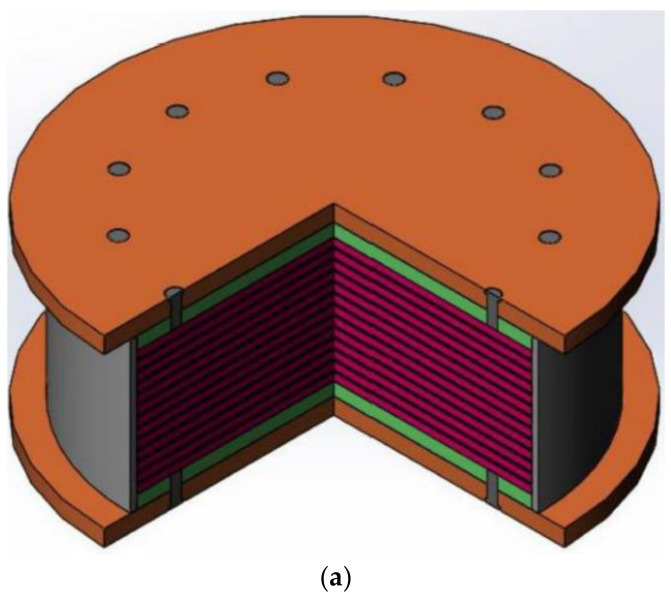

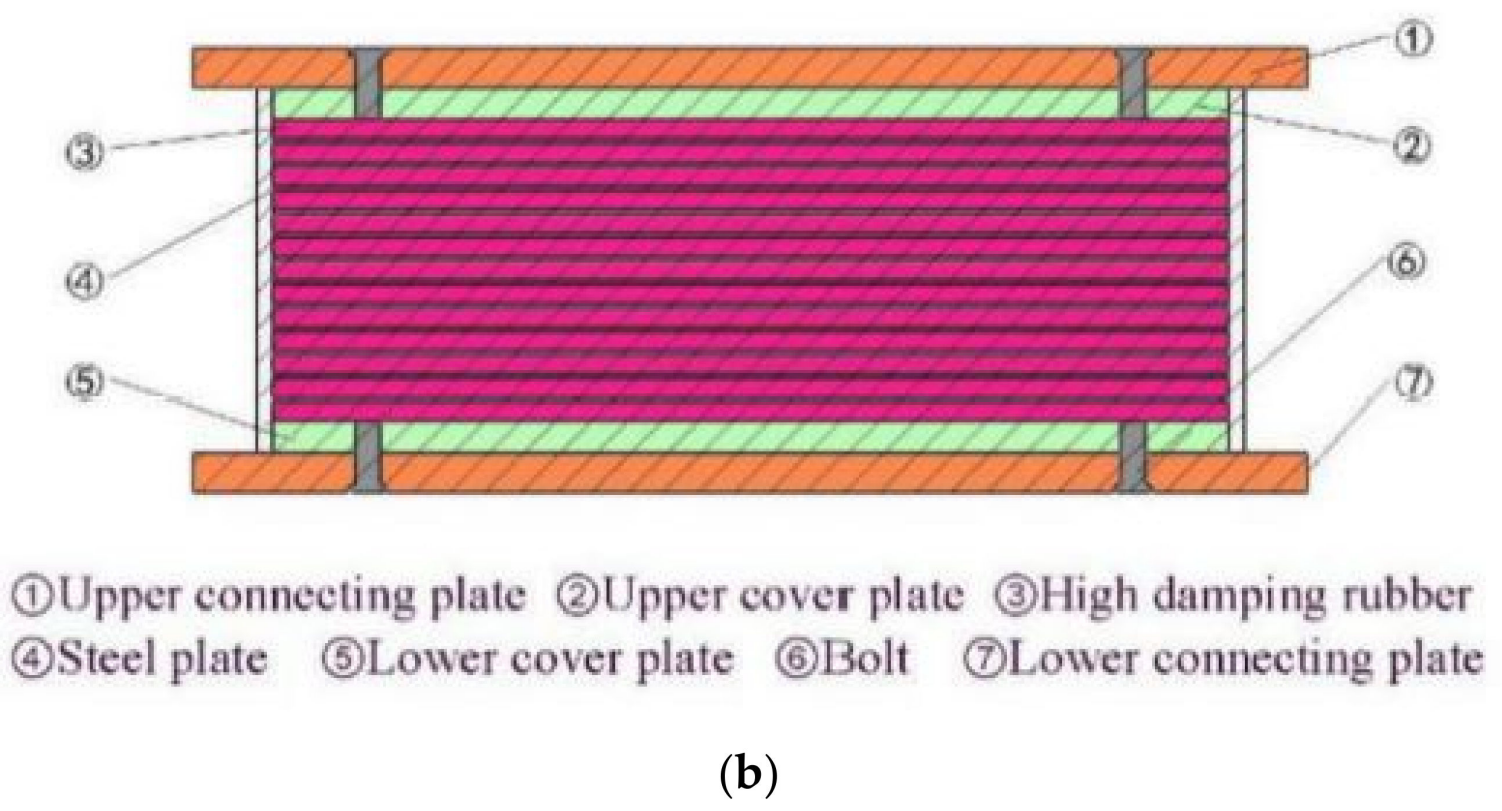
Schematic illustration of the high-damping rubber isolation bearing: (**a**) 1/4 perspective view in 3D and (**b**) Cross-section view [7].

High-damping rubber isolation bearings consist of alternating high-damping rubber layers bonded between laminated thin steel plates, which are vulcanized at high temperatures and pressures, as shown in Figure 1 [7,8]. They have a wider perspective of applications in building structures, and their isolation performance mainly depends on the mechanical and damping energy dissipation characteristics of the high-damping rubber material. Therefore, designing high-damping rubber materials with excellent vertical performance is key to effectively controlling and reducing the vibration of rail transit systems transmitted into building structures.

High-damping rubber materials present features of hyperelasticity and viscoelasticity. Considering the numerical stability and the physical plausibility of mechanical behaviors, a hyperelastic model, which satisfied the Baker–Ericksen inequality, was recently proposed [9]. It was shown that the model could accurately capture the hyperelastic behavior of isotropic rubber-like materials both in homogeneous and non-homogeneous deformation under uniaxial tensile tests. A finite element implementation of hyperelastic polymeric materials was reported using three hyperelastic models, including Biderman, power-law, and exponential-linear law models [10]. To the best knowledge of the authors, the hyper-viscoelastic behaviors of the high-damping rubber material used in high-damping rubber isolation bearings during the cyclic tension and compression process in the vertical direction are still not sufficiently understood. Uniaxial and biaxial tensile tests, cyclic shear, and stress relaxation tests were conducted on different types of high-damping rubber materials used for laminated rubber bearings [11,12,13]. Based on the experimental results, a constitutive model was proposed, which combined the hyperelastic behavior with damage and the elastoplastic behavior with the strain-dependent isotropic hardening of rubber materials without considering the viscoelastic effect of the material. A constitutive model was proposed using the theory of pseudo-elasticity for the quasi-static loading-unloading of filler-reinforced rubbers (filled with 60 parts of carbon black per hundred rubber), which accounted for stress softening and residual strain effects [14]. A constitutive model of the hyper-viscoelastic materials, combining the Ogden model with the generalized Maxwell model, was reported and validated under relaxation tests of uniaxial tension with different loading histories [15]. Another hyper-viscoelastic model, combining the polynomial strain energy density function of material hyperelasticity with the generalized Maxwell model, was proposed. It was shown that using this model could obtain a high accuracy for the prediction of the mechanical behavior of uniaxial tension and short-term tension creep, while the cyclic tension and compression process had not been considered [16]. Additionally, a principle-stretch-based micro-macro constitutive model, considering the nonlinear viscosity and microstructural features of VHB4910, HNBR50, and carbon black-filled elastomers, was established and validated under both uniaxial tensile and cyclic tension-compression tests [17,18,19]. It was demonstrated that this constitutive model was capable of characterizing the material properties and giving a good quantitative prediction of the Mullins effect. On this basis, a finite element framework was further established when attempting to predict the viscoelastic behavior of elastomers, particularly when an elastomer was undergoing a non-uniform deformation [20]. To describe the nonlinear viscoelastic behavior of the acrylonitrile butadiene rubber (NBR)/polyvinyl chloride (PVC) rubber blend reinforced by nanoclay and graphene, the Marlow hyperelastic model was used combining with the strain hardening power-law model and Ogden and Roxburgh function to describe Mullins effect [21]. It was shown that this model could predict well the cyclic tension and compression process of the material. However, these hyper-viscoelastic constitutive models have not considered the volumetric deformation caused by the material compressibility and the anisotropic change in the material properties due to Mullins and the residual strain effects in the vertical direction during the cyclic tension and compression process.

This paper aims to investigate the hyper-viscoelastic properties of the high-damping rubber material during the cyclic tension and compression process in the vertical direction. These properties include hyperelastic parameters, viscoelastic coefficients, and the relaxation time of the rubber material. To this end, uniaxial cyclic tension and compression tests were conducted, during which the Mullins and residual strain effects were observed. Secondly, the three-element Maxwell rheological model, combining a strain energy density function describing the material hyperelasticity, was proposed for modeling the hyper-viscoelastic behaviors of the high-damping rubber material during the cyclic tension and compression process according to works [11,22,23]. Based on the obtained results, an iterative identification procedure [24,25] was used to determine the constitutive parameters of the material for each loading-unloading cycle. Identified parameters were further expressed as a function of the number of cycles. Experimental characterizations provide a basis for the optimal design of gradients in this rubber material. The findings in this work provide an outlet for the explanation of the variations in the vertical stiffness and the damping energy dissipation of high-damping rubber isolation bearings during the cyclic loading-unloading process in practical engineering applications: (a) the volumetric deformation caused by the material compressibility and (b) the anisotropic effect due to Mullins and residual strain effects in the vertical direction.

The motivation of this work was to characterize the hyper-viscoelastic properties of the high-damping rubber material for each loading-unloading cycle during the cyclic tension and compression process, during which the Mullins and residual strain effects were observed. The characterized hyper-viscoelastic properties would facilitate the controlling and optimization of the isolation performance of the high-damping rubber material in the vertical direction used for high-damping rubber isolation bearings, which have a wider perspective of applications in the urban rail transit system.

## 2. Experimental Characterization

**Figure 2:**
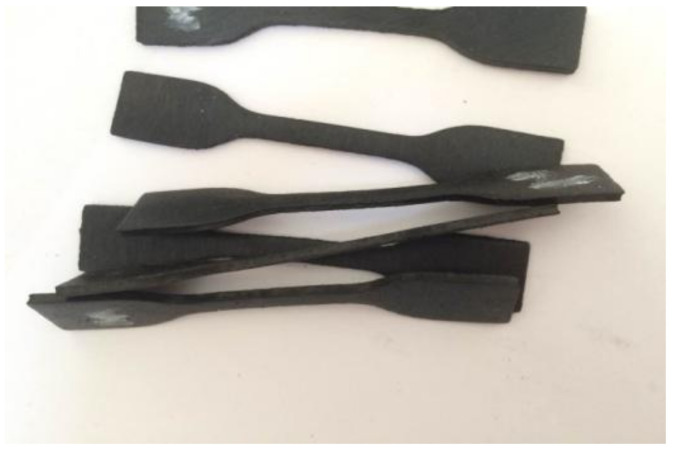
Dumbbell-shaped specimens of the high-damping rubber material for tension tests.

**Figure 3:**
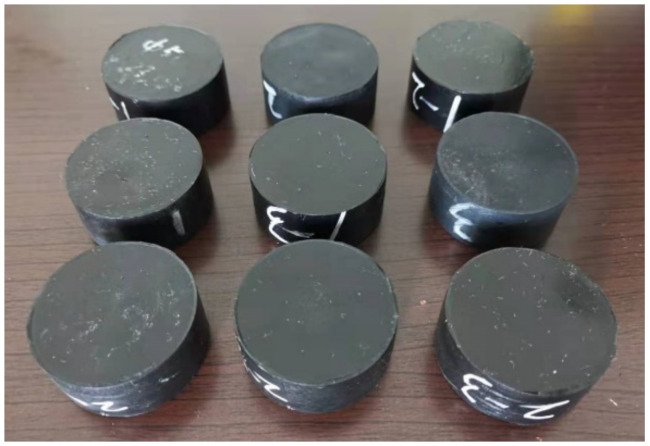
Cylindrical specimens of the high-damping rubber material for compression tests.

**Figure 4:**
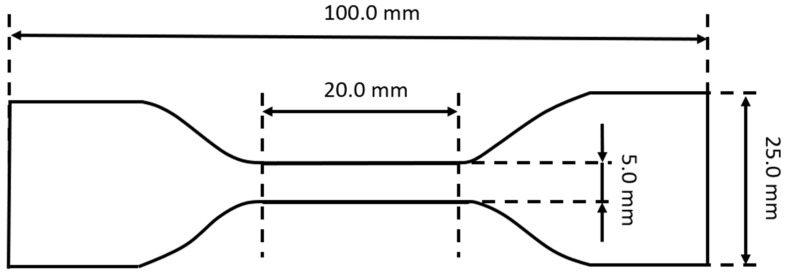
Schematic representation of dumbbell-shaped specimens with dimensions.

To characterize the hyper-viscoelastic properties of the high-damping rubber material, loading-unloading cyclic tension and compression tests were carried out. To ensure the reproducibility of the results, cyclic tension and compression tests were, respectively, repeated three times using three different specimens, and the average value of the obtained results of each test was used to represent the final experimental result. The obtained experimental data were used for the inverse identification of hyper-viscoelastic constitutive parameters of the high-damping rubber material. This was according to the relevant requirements of Chinese Standard JT/T 842-2012 [26]. The dumbbell-shaped and cylindrical specimens of the high-damping rubber material were, respectively, prepared for uniaxial cyclic tension and compression tests, are shown in Figure 2 and Figure 3. The dimensions of the dumbbell-shaped specimen have been schematically illustrated in Figure 4, and the cylindrical specimens have dimensions of 29 mm × 12.5 mm (diameter × height). These specimens were provided by the manufacturing company of high-damping rubber isolation bearings, which is in cooperation with the Institute of Engineering Mechanics, China Earthquake Administration (IEM, CEA). The basic formulation of the rubber material was shown in Table 1. Due to commercial confidentiality, no further information about the ingredients and the production process of this rubber material could be provided.

**Figure 5:**
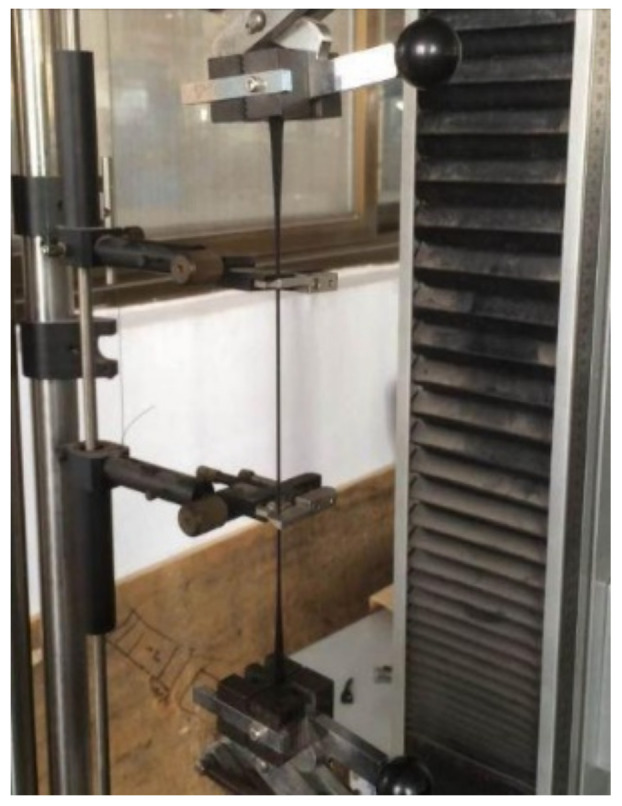
Tension testing machine.

**Figure 6:**
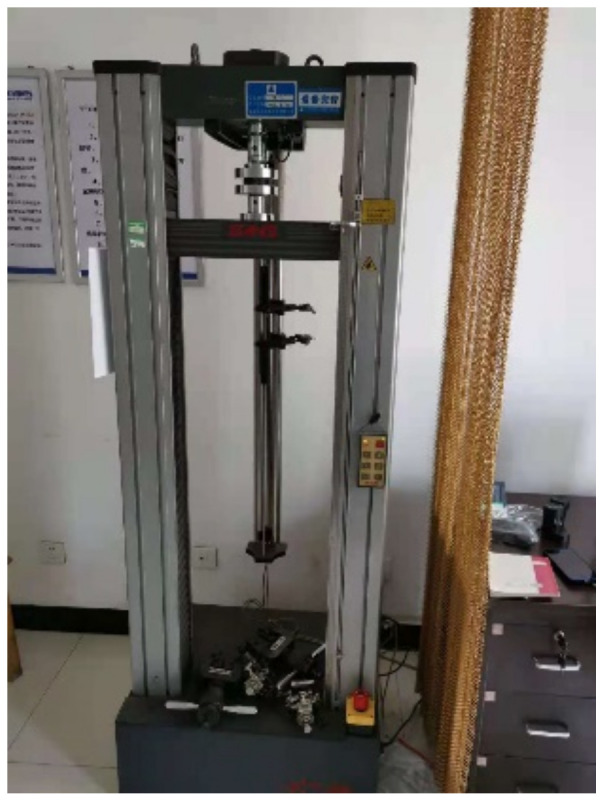
Compression testing machine.

It is assumed that the material undergoes uniaxial uniform deformation during the test, and the effect of temperature variation on the rubber material is not considered in this work. According to the Chinese Standard GB/T 20688.1-2007 [27], tests should be achieved under an ambient temperature. Cyclic tension tests were performed using a tension testing machine, as shown in Figure 5, at a loading rate of 10 mm/min and with a maximum stretch ratio of 250%. Using the compression testing machine, as shown in Figure 6, the loading rate of 1 mm/min was applied to perform cyclic compression tests on three specimens, respectively, and the maximum compression reached 50% of the original height. The loading-unloading cycle was repeated five times during the cyclic and compression process, and the unloading rate was kept the same as the loading one. Evolutions of the stress and the strain with respect to time were obtained, respectively.

## 3. Constitutive Model

High-damping rubber materials are hyper-viscoelastic materials, which exhibit hyperelasticity and viscoelasticity under cyclic loading conditions, and the viscoelasticity enables the high-damping rubber material to possess the capability of damping energy dissipation. The hyper-viscoelastic properties of high-damping rubber materials determine the characteristics of the elastic reset and the damping energy dissipation of high-damping rubber isolation bearings. By assuming that the high-damping rubber material is incompressible, the strain energy density function describing the material hyperelasticity can be reduced to its isochoric part, which reads as follows [11]:

(1) $$\bar{W}=c_{4}\left({\bar{I}}_{1}-3\right)+c_{5}\left({\bar{I}}_{2}-3\right)+\frac{c_{4}c}{m+1}{\left(\frac{{\bar{I}}_{1}-3}{c}\right)}^{m+1}$$

where $c_{4}$, $c_{5}$, c and m are material parameters; ${\bar{I}}_{1}$, ${\bar{I}}_{2}$ are, respectively, the first and the second invariants of isochoric right Cauchy–Green tensor, and are expressed as follows:

(2) $$\left\{\begin{array}{l} {\bar{I}}_{1}=\mathrm{tr}\left(\bar{C}\right)\\ {\bar{I}}_{2}=\frac{1}{2}\left[{\left(\mathrm{tr}\left(\bar{C}\right)\right)}^{2}-\mathrm{tr}\left({\bar{C}}^{2}\right)\right] \end{array}\right.$$

where $\bar{C}=J^{-\frac{2}{3}}F^{T}F$ is the isochoric right Cauchy–Green tensor; ${\bar{C}}^{-1}=J^{\frac{2}{3}}F^{-1}F^{-T}$ is the inverse of the isochoric right Cauchy–Green tensor; F is the deformation gradient tensor and $J=\det\left(F\right)$ is the determinant of the deformation gradient tensor.

**Figure 7:**
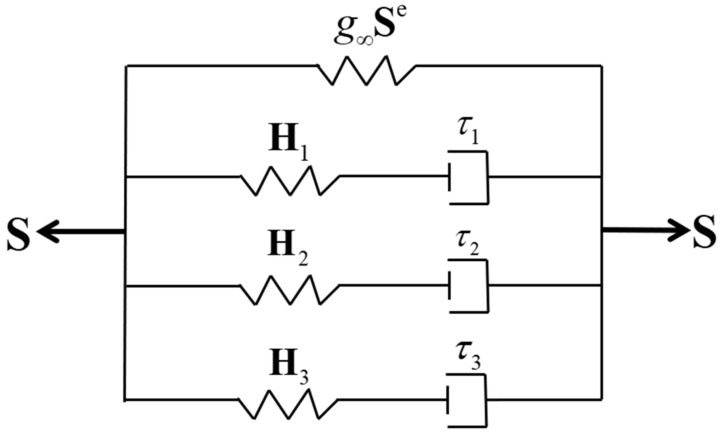
Three-element Maxwell relaxation model.

Due to the rheological characteristics of high-damping rubber materials, it needs to introduce the time effect on the material hyperelasticity to describe the viscoelastic behavior of the high-damping rubber material. In this work, a three-element Maxwell relaxation model was used to describe the hyper-viscoelastic behavior of the rubber material, as shown in Figure 7. In the model, the second Piola–Kirchhoff total stress tensor S is the sum of the elastic component $g_{\infty }S^{e}$ and the viscoelastic overstress tensor $H_{j}$(j=1,2,3), which is written as follows [22]:

(3) $$S=g_{\infty }S^{e}+\sum_{j=1}^{3}H_{j}$$

with

(4) $$\left\{\begin{array}{l} S^{e}=2\frac{\partial \bar{W}}{\partial \bar{C}}-\frac{2}{3}\left(\frac{\partial \bar{W}}{\partial \bar{C}}:\bar{C}\right){\bar{C}}^{-1}\\ H_{j}=\int_{0}^{t}g_{j}e^{-\frac{t-\tau }{\tau_{j}}}\frac{d}{dt}\left(S^{e}\right)d\tau \;\left(j=1,2,3\right) \end{array}\right.$$

where t is the time; $S^{e}$ is the instantaneous second Piola–Kirchhoff elastic stress tensor; $g_{\infty }$ is the viscoelastic coefficient characterizing the long-term elastic response; $g_{j}$ and $\tau_{j}$ (j=1,2,3) are, respectively, the viscoelastic coefficient characterizing the viscous response and the relaxation time corresponding to the j-th Maxwell element.

To identify the constitutive parameters involved in the model, three-dimensional hyper-viscoelastic constitutive equations should be decoupled and formulated into one-dimensional ones, which are suitable for parameter identification. Considering the incompressibility of the high-damping rubber material and assuming that the material specimen does not undergo rigid translational motion, the relation between the current configuration x and the initial configuration X of the material reads as follows:

(5) x=FX

In the case of a specimen subjected to uniaxial tension and compression, the deformation gradient tensor F takes the form as follows:

(6) $$F=\left[\begin{array}{lll} \lambda & 0 & 0\\ 0 & \lambda^{-\frac{1}{2}} & 0\\ 0 & 0 & \lambda^{-\frac{1}{2}} \end{array}\right]$$

where λ>1 is the principle stretch ratio.

Combining Equations (1)–(6) and using the Taylor numerical integration algorithm [28] to discretize the Equation (4) [24,29], one-dimensional constitutive equations used for the iterative identification procedure of material parameters, which will be presented in the next section, can be summarized as follows:

(7) $$\left\{\begin{array}{c} S_{11}\left(t_{n+1}\right)=g_{\infty }S_{11}^{e}\left(t_{n+1}\right)+\sum_{j=1}^{N}H_{j}\left(t_{n+1}\right)\\ H_{j}\left(t_{n+1}\right)=e^{-\frac{\Delta t}{\tau_{j}}}H_{j}\left(t_{n}\right)+g_{j}\frac{1-e^{-\frac{\Delta t}{\tau_{j}}}}{\frac{\Delta t}{\tau_{j}}}\left[S_{11}^{e}\left(t_{n+1}\right)-S_{11}^{e}\left(t_{n}\right)\right]\\ S_{11}^{e}\left(t_{n+1}\right)=2p\left(t_{n+1}\right)-2c_{5}\lambda^{2}\left(t_{n+1}\right)-\frac{2}{3}\frac{p\left(t_{n+1}\right)}{\lambda^{2}\left(t_{n+1}\right)}{\bar{I}}_{1}\left(t_{n+1}\right)+\frac{2}{3}c_{5}\left(\lambda^{2}\left(t_{n+1}\right)+\frac{2}{\lambda^{4}\left(t_{n+1}\right)}\right)\\ T_{11}\left(t_{n+1}\right)=\lambda^{2}\left(t_{n+1}\right)S_{11}\left(t_{n+1}\right) \end{array}\right.$$

with

(8) $$p\left(t_{n+1}\right)=c_{4}\left[1+{\left(\frac{{\bar{I}}_{1}\left(t_{n+1}\right)-3}{c}\right)}^{m}\right]+c_{5}{\bar{I}}_{1}\left(t_{n+1}\right)$$

where $S_{11}$ is the first component of the second Piola–Kirchhoff total stress tensor; $S_{11}^{e}$ is the first component of the instantaneous second Piola–Kirchhoff elastic stress tensor; $H_{j}$(j=1,2,3) is the viscoelastic overstress; $T_{11}$ is the first component of the first Piola–Kirchhoff total stress tensor; $t_{n}$ and $t_{n+1}$ are, respectively, the *n*-th and (*n* + 1)-th time instant and $\Delta t=t_{n+1}-t_{n}$ is the time step.

## 4. Iterative Identification Procedure

To assess the predictive capabilities of the constitutive model proposed in this work, an iterative identification procedure was carried out to determine the constitutive parameters for different loading-unloading cycles. To this end, for each cycle, an objective function was defined as follows [22,24,25]:

(9) $${ℜ}_{p_{i}}\left(\Omega \right)=\sum_{j=1}^{N_{p_{i}}}{\left\{\left[{\left(T_{11}\left(\Omega \right)\right)}_{j}^{\mathrm{model}}-{\left({\tilde{T}}_{11}\right)}_{j}^{\exp}\right]\right\}}^{2}$$

where $\Omega ={\left[c_{4},c_{5},c,m,g_{\infty },g_{1},g_{2},g_{3}\right]}^{T}$ is the space vector of the constitutive parameters of the material; $N_{p_{i}}$ is the number of experimental data measured during the loading-unloading cycle; ${\left(T_{11}\left(\Omega \right)\right)}_{j}^{\mathrm{model}}$ is the j-th model value of the first component of the first Piola–Kirchhoff total stress tensor, and ${\left({\tilde{T}}_{11}\right)}_{j}^{\exp}$ is the associated experimental value.

Parameters in the space vector Ω satisfies:

(10) $$\left\{\begin{array}{l} c_{4}\geq 0,\;c_{5}\geq 0,\;c\geq 0,\;m\geq 0\\ 0\leq g_{\infty }<1\\ 0\leq g_{i}<1,\;i=1,2,3 \end{array}\right.$$

Using pattern search algorithm built-in MATLAB software [30], viscoelastic coefficients with their initial values in the optimization procedure satisfies:

(11) $$\left\{\begin{array}{l} g_{\infty }+\sum_{i=1}^{3}g_{i}=1\\ g_{\infty }^{o}=g_{1}^{o}=g_{2}^{o}=g_{3}^{o}=0.25 \end{array}\right.$$

Note that “o” denotes the initial value of viscoelastic coefficients at the beginning of the iteration step in the optimization procedure.

The relaxation time corresponding to each Maxwell element should satisfy the conditions as follows [24]:

(12) $$\tau_{i}\geq 0,\;i=1,2,3$$

According to the work [24], within the range $\left[\tau_{\min},\tau_{\max}\right]$, the values of relaxation time used in the first iterative identification procedure read as follows:

(13) $$\left\{\begin{array}{l} \tau_{1}^{\left(0\right)}=\tau_{\min}\\ \tau_{2}^{\left(0\right)}=\tau_{\min}10^{\Delta }\\ \tau_{3}^{\left(0\right)}=\tau_{\max} \end{array}\right.$$

where $\tau_{\min}$ is set equal to the sampling interval; $\tau_{\max}$ is set equal to the total time of one loading-unloading cycle and Δ is the logarithmic time step, which is defined as follows:

(14) $$\Delta =\frac{1}{2}\log\frac{\tau_{\max}}{\tau_{\min}}$$

After the first iteration in the iterative identification procedure, if the coefficient of the determination $R^{2}$ is smaller than the set threshold $R_{\mathrm{th}}^{2}$, using the parameters obtained by the optimization procedure, pattern search algorithm, and the values of relaxation time in the Equation (13), then the relaxation time is split according to the following rule in the *j*-th iteration (*j* ≥ 2):

(15) $$\tau_{i}^{\left(j\right)}\Rightarrow \tau_{i}^{\left(j\right)}\left\{10^{-{\left(\frac{\Delta }{3}\right)}^{j}},\;1,\;10^{{\left(\frac{\Delta }{3}\right)}^{j}}\right\},i=1,2,3$$

Accordingly, it needs twenty-seven times for the optimization procedure of one iteration in the iterative identification procedure. If the maximum coefficient of determination among those optimization procedures is still smaller than the set threshold, then it proceeds to the (*j* + 1)-th iteration until obtaining the set of parameters enabling $R^{2}$ to be larger than $R_{\mathrm{th}}^{2}$. At this moment, the obtained set of parameters is the optimal set of parameters.

## 5. Results and Discussion

**Figure 8:**
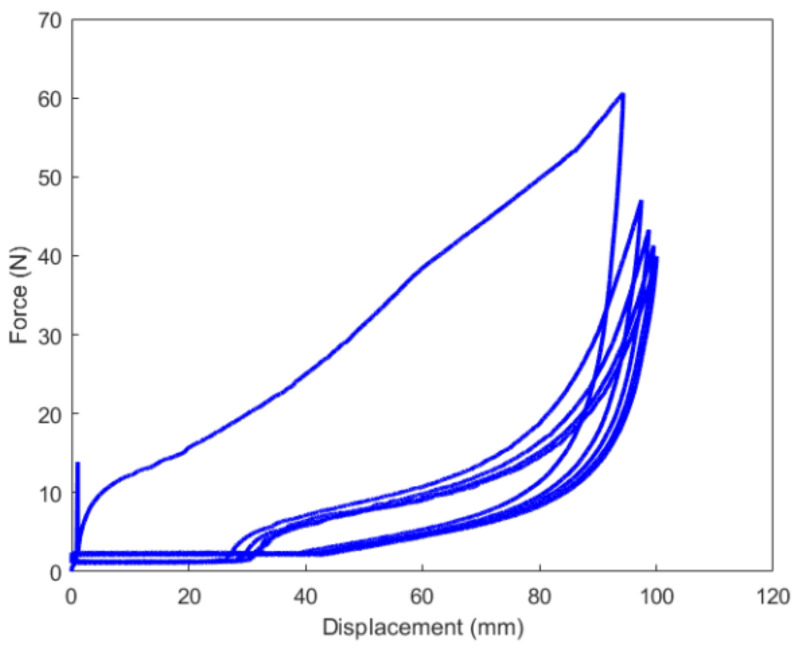
Result of the cyclic tension process.

**Figure 9:**
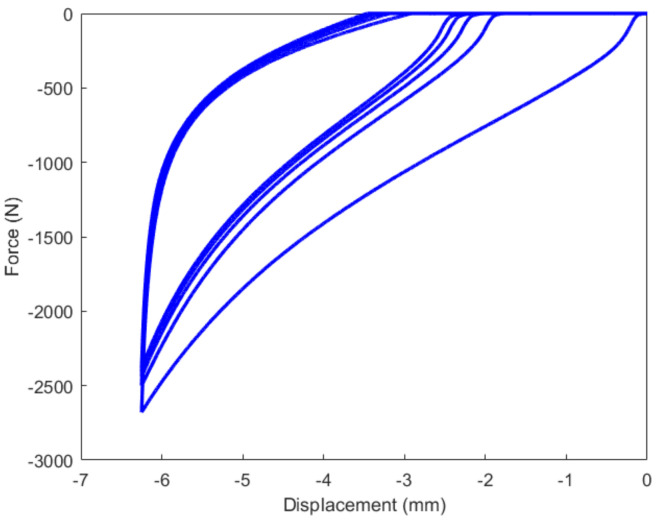
Result of the cyclic compression process.

The results of the cyclic tension and compression process have been respectively illustrated in Figure 8 and Figure 9. From the results, the high-damping rubber material prepared in this work exhibits a characteristic of the large deformation of elastomers with a marked hysteretic response during the loading-unloading cycle. At the same strain level, the stress on the unloading path is significantly less than that of the loading one. The difference in the stresses corresponding to the same strain level under loading and unloading primarily depends on the proportion of filler in the rubber compound [14]. It is the greatest during the first cycle and gradually stabilizes after a number of cycles. Furthermore, the stress softening associated with Mullins and residual strain effects have been observed during both the cyclic tension and compression process.

**Figure 10:**
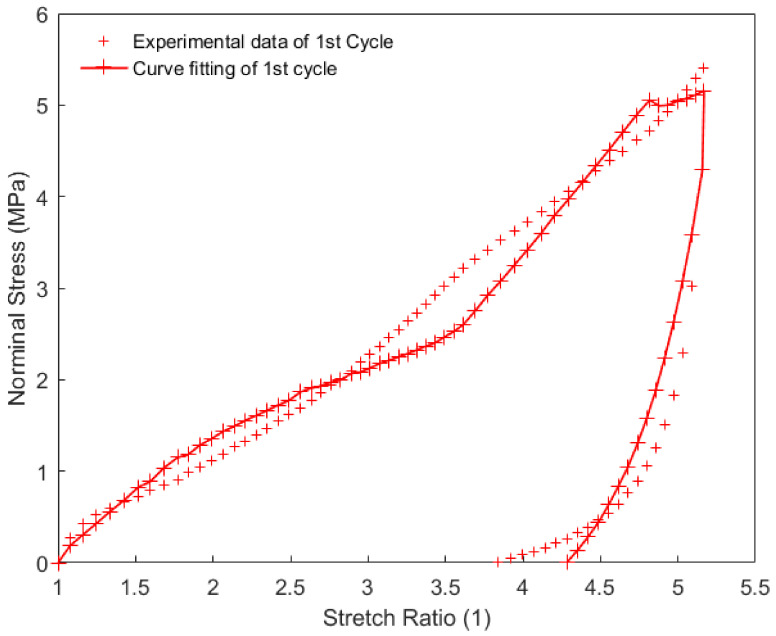
Model prediction of the first cycle during the cyclic tension process of the high-damping rubber material.

**Figure 11:**
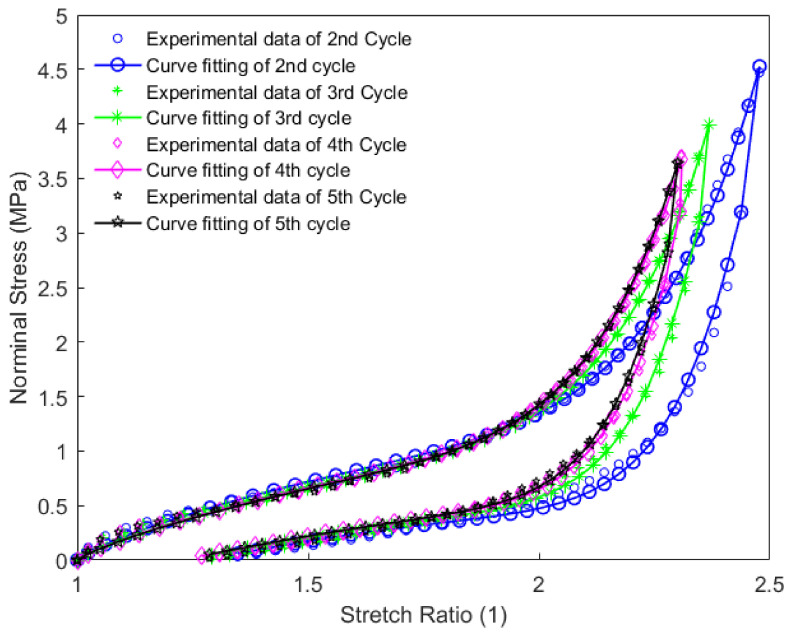
Model prediction of the second to the fifth cycle during the cyclic tension process of the high-damping rubber material.

**Figure 12:**
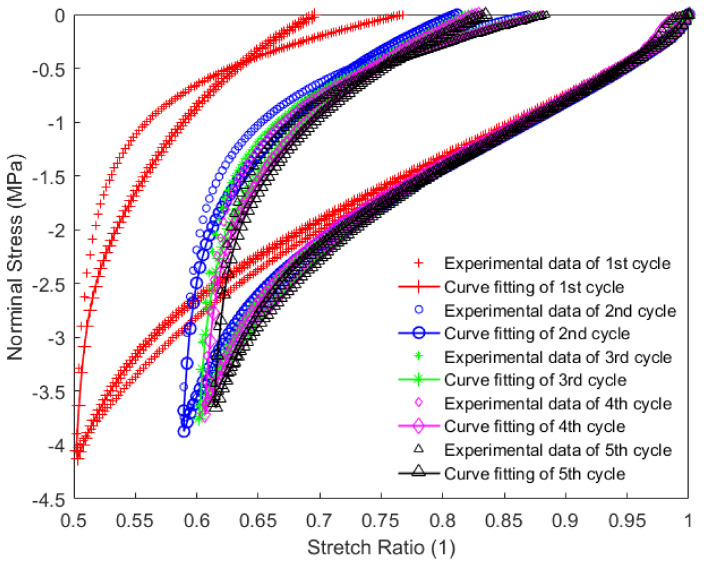
Model prediction of different cycles during the cyclic compression process of the high-damping rubber material.

In this work, each loading-unloading cycle during the cyclic tension and compression process was independently extracted from the results. They were expressed as an evolution of the nominal stress with respect to the corresponding stretch ratio, in which the nominal stress was calculated as the applied force divided by the initial cross-sectional area. Constitutive parameters of the material were identified using the iterative identification procedure presented in Section 4. The pattern search algorithm built-in MATLAB R2016a was used for minimizing the objective function in the optimization procedure. The obtained parameters for each loading-unloading cycle are summarized in Table 2. The model results of the cyclic tension and the compression of the material using these parameters for each loading-unloading cycle were compared with the obtained experimental findings, as shown in Figure 10, Figure 11 and Figure 12. The values of $R^{2}$ for each loading-unloading cycle are listed in Table 3. It is observed that the constitutive model used in this work well represents the hyper-viscoelastic behavior of the high-damping rubber material in the vertical direction from the second to the fifth cycle during the cyclic tension process, while a mismatch was observed between the curve fitting and the experimental data for the first cycle during the cyclic tension process and all cycles during the cyclic compression process. This could be explained due to the fact that, in the actual situation, the rubber materials might suffer significantly from a volumetric deformation during the first cycle, and afterward, the isochoric deformation becomes more predominant during the subsequent cycles of the cyclic tension process, such that the volumetric deformation is negligible. During the cyclic compression process, the effect of the volumetric deformation is not trivial, and it could also contribute to affecting the hyper-viscoelastic behavior of the material. Nevertheless, it was assumed that the rubber materials were incompressible in the constitutive model.

It can be judged from Figure 10 and Figure 11 that the residual strain of the first cycle during the cyclic tension process was much larger than that of the other cycles. With the increase in the number of cycles, the residual strain remained almost unchanged. Figure 12 indicates that with the increase in the number of cycles during the cyclic compression process, the residual strain gradually decreases. This phenomenon may also probably increase the vertical stiffness of the rubber material together with the Mullins effect.

**Figure 13:**
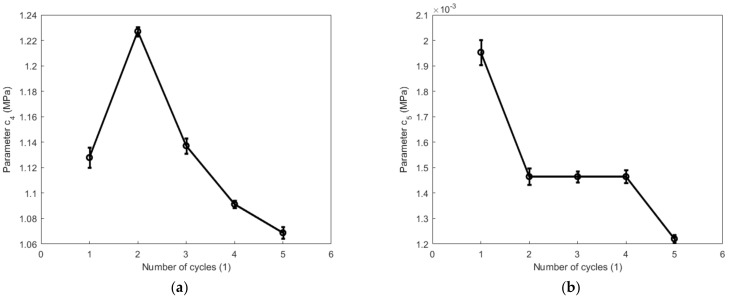

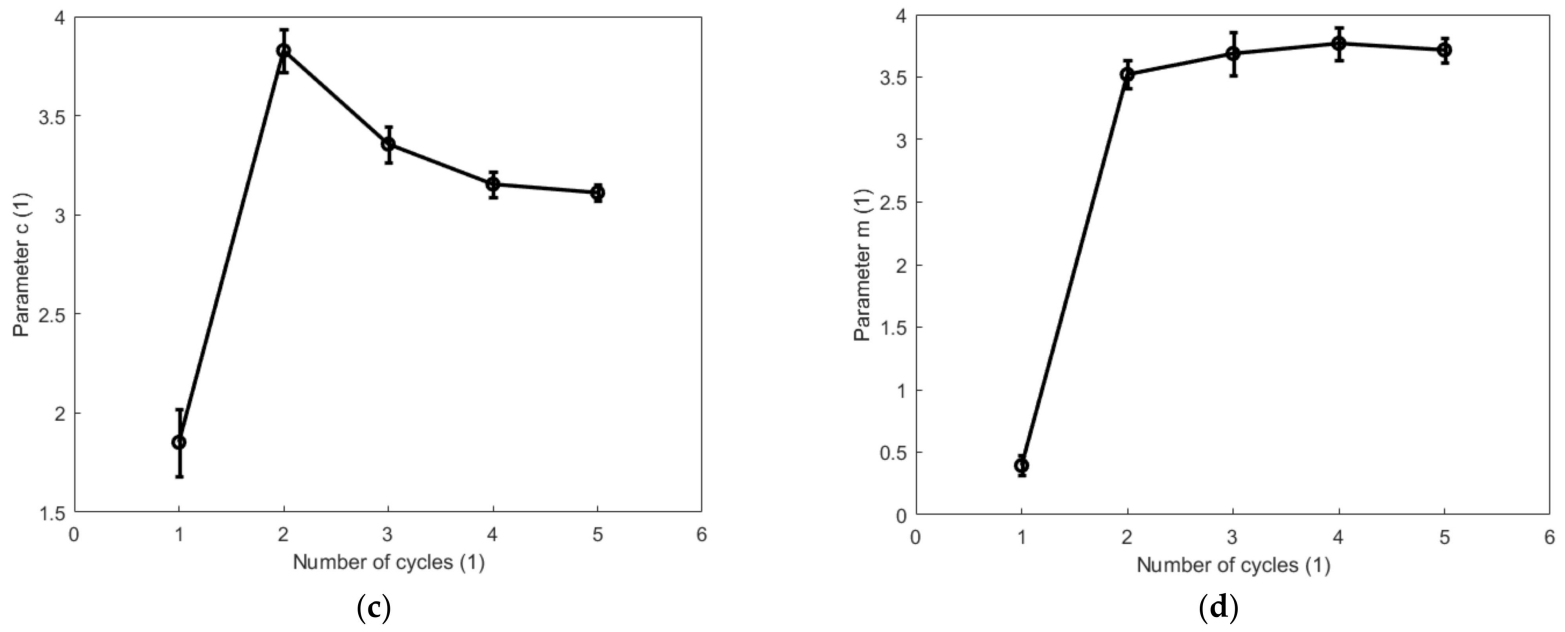
Evolutions of parameters $c_{4}$, $c_{5}$, c, and m with respect to the number of cycles during the cyclic tension process: (**a**) $c_{4}$; (**b**) $c_{5}$; (**c**) c, and (**d**) m.

**Figure 14:**
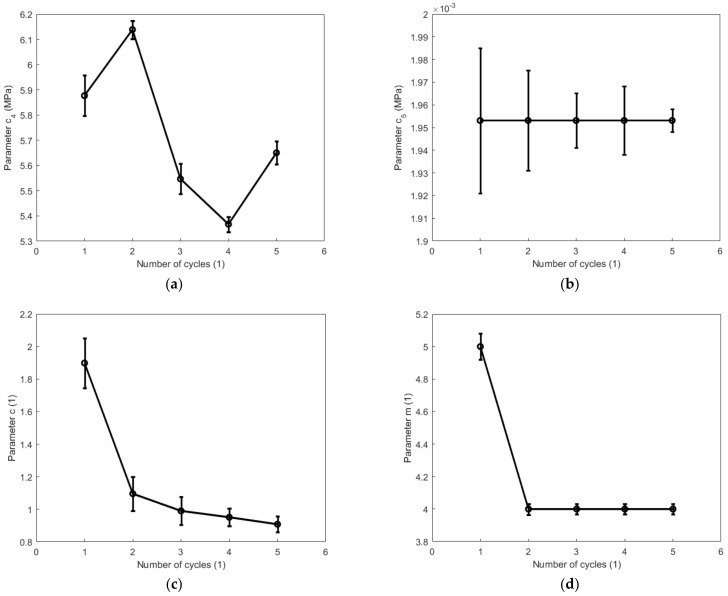
Evolutions of parameters $c_{4}$, $c_{5}$, c, and m with respect to the number of cycles during the cyclic compression process: (**a**) $c_{4}$; (**b**) $c_{5}$; (**c**) c, and (**d**) m.

Taking into account the impact of Mullins and the residual strain effects on hyper-viscoelastic properties of the high-damping rubber material when the material was degraded by the cyclic tension and compression process, the constitutive parameters presented in Table 2 were expressed as a function of the number of cycles. The evolutions of parameters $c_{4}$, $c_{5}$, c and m describing the hyperelasticity of the high-damping rubber material with respect to the number of cycles during the cyclic tension and compression process are, respectively, illustrated in Figure 13 and Figure 14. From these figures, the parameter $c_{4}$ during the cyclic tension process gradually decreases with the number of cycles after the second cycle, while it fluctuates significantly during the cyclic compression process. The maximum ratio of variation for the parameter $c_{4}$ reaches as high as 13% during both the cyclic tension and compression process. The parameter $c_{5}$ is negligible compared with the other hyperelastic parameters during the tension and compression process. The parameter m is mainly affected by the first cycle and is weakly impacted afterward. As judged from the parameters $c_{4}$ and $c_{5}$, the cyclic tension and compression process of the high-damping rubber material is mainly impacted by the first invariant instead of the second invariant of the isochoric right Cauchy-Green tensor.

**Figure 15:**
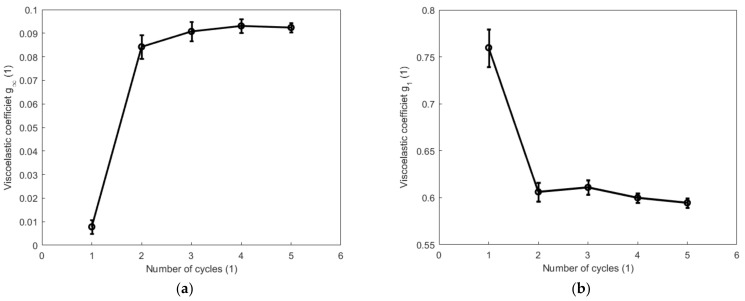

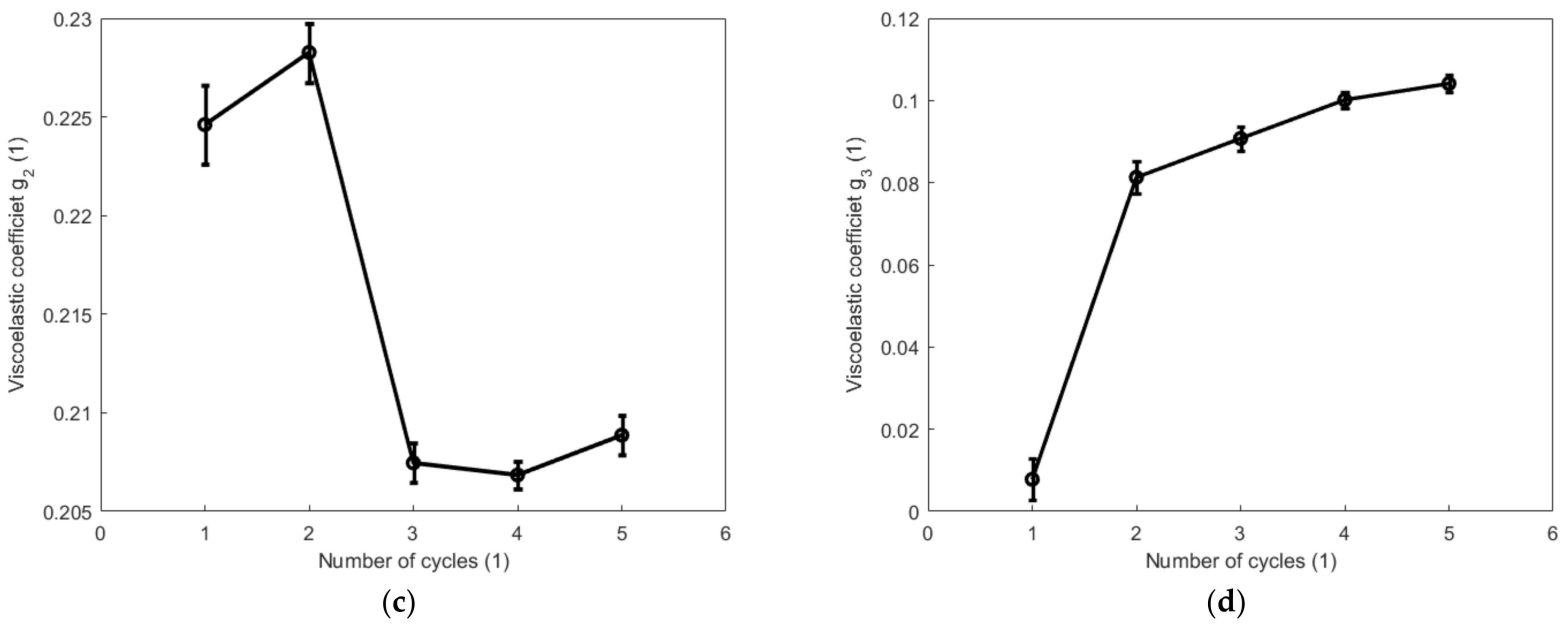
Evolutions of viscoelastic coefficients $g_{\infty }$, $g_{1}$, $g_{2}$, and $g_{3}$ with respect to the number of cycles during the cyclic tension process: (**a**) $g_{\infty }$; (**b**) $g_{1}$; (**c**) $g_{2}$, and (**d**) $g_{3}$.

**Figure 16:**
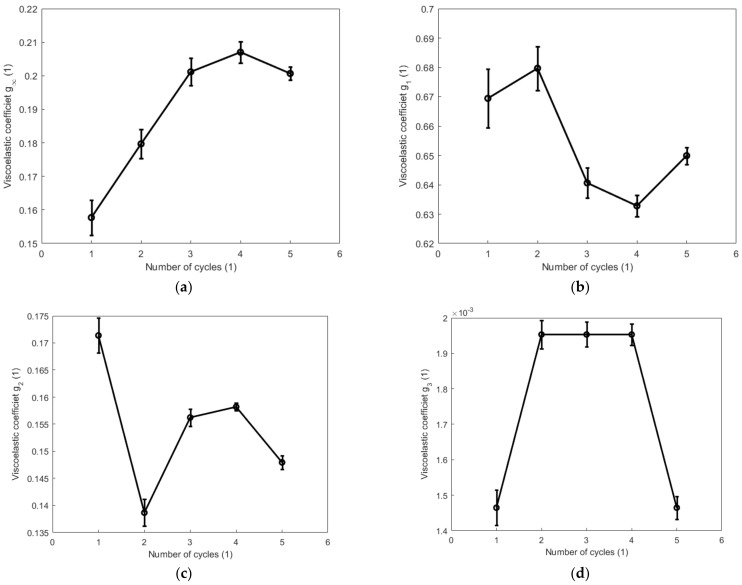
Evolutions of viscoelastic coefficients $g_{\infty }$, $g_{1}$, $g_{2}$, and $g_{3}$ with respect to the number of cycles during the cyclic compression process: (**a**) $g_{\infty }$; (**b**) $g_{1}$; (**c**) $g_{2}$, and (**d**) $g_{3}$.

**Figure 17:**
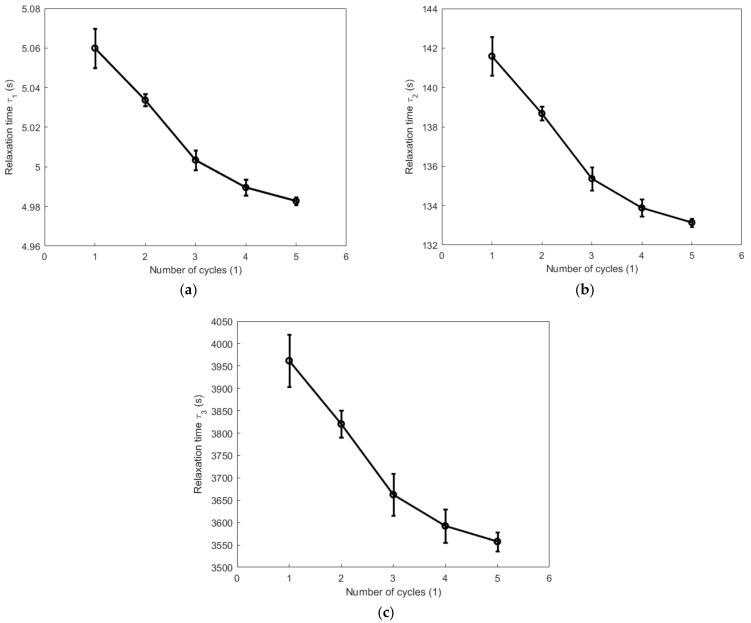
Evolutions of relaxation times $\tau_{1}$, $\tau_{2}$, and $\tau_{3}$ with respect to the number of cycles during the cyclic tension process: (**a**) $\tau_{1}$; (**b**) $\tau_{2}$, and (**c**) $\tau_{3}$.

**Figure 18:**
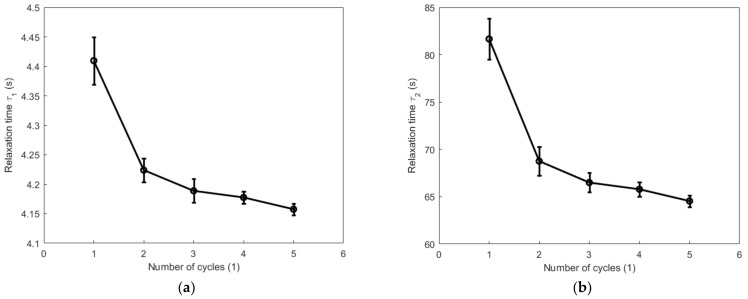

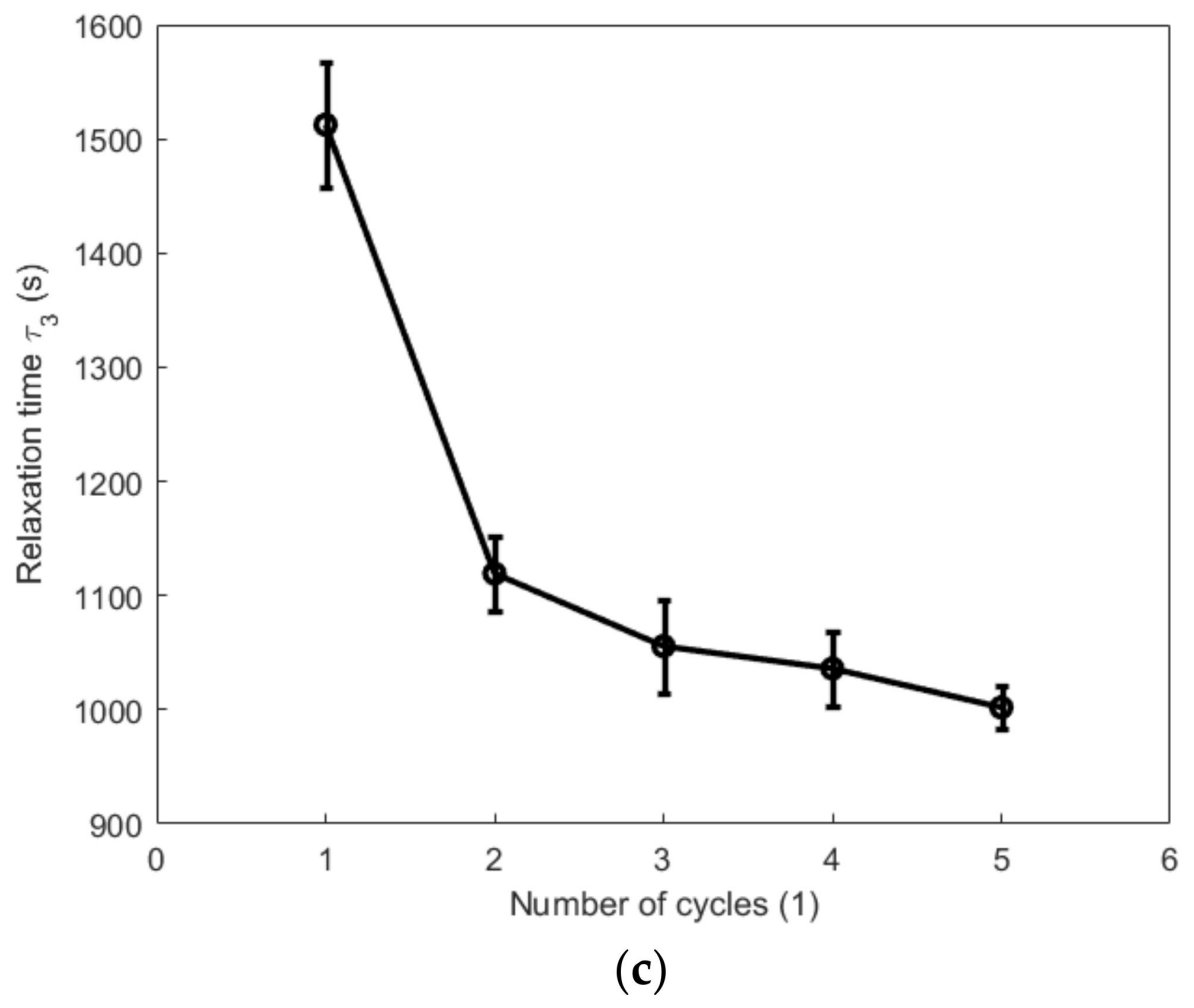
Evolutions of relaxation times $\tau_{1}$, $\tau_{2}$, and $\tau_{3}$ with respect to the number of cycles during the cyclic compression process: (**a**) $\tau_{1}$; (**b**) $\tau_{2}$, and (**c**) $\tau_{3}$.

Figure 15 and Figure 16 show, respectively, the evolutions of the viscoelastic coefficients $g_{\infty }$, $g_{1}$, $g_{2}$, and $g_{3}$ with respect to the number of cycles during the cyclic tension and compression process. Coefficient $g_{\infty }$ determines the long-term response of the elastic behavior of the material. It increases with the increase in the number of cycles and reaches its maximum during the fourth cycle for both the cyclic tension and compression process. Coefficient $g_{1}$ generally shows a decreasing trend during both the cyclic tension and compression process, and it decreases more evidently during the cyclic tension process than in the cyclic compression process. Coefficient $g_{2}$ decreases significantly after the second cycle during the cyclic tension process. During the cyclic compression process, it sharply decreases by 17.6% after the first cycle and increases after the second cycle. The value of coefficient $g_{3}$ obviously augments during the cyclic tension process, while it remains almost unchanged at a comparatively lower magnitude during the compression process. Figure 17 and Figure 18 show, respectively, the evolutions of the relaxation times $\tau_{1}$, $\tau_{2}$, and $\tau_{3}$ with respect to the number of cycles during the cyclic tension and compression process. The results show that $\tau_{1}$, $\tau_{2}$, and $\tau_{3}$ simultaneously decrease with the number of cycles during both the cyclic tension and compression process. As judged from the results above, during the cyclic tension and compression process, the elastic response of the material is gradually enhanced, and its viscous response exhibits a decreasing trend. This further indicates that there exists a hardening effect in this high-damping rubber material during the cyclic tension and compression process. Moreover, it was further found that the material hardens much faster during the cyclic compression process than the cyclic tension process. This discrepancy is probably caused by the nonlinear viscosity change in the elastomers when the rubber material undergoes a different deformation mode. This hardening may probably stabilize after the fifth cycle, as judged from the decreasing trend of the evolutions of the relaxation time with respect to the number of cycles.

## 6. Conclusions and Perspectives

In this work, the hyper-viscoelastic properties of the high-damping rubber material were characterized for each loading-unloading cycle during the cyclic tension and compression process. To this end, uniaxial cyclic tension and compression tests were carried out, in which the Mullins and residual strain effects were observed during the process. Secondly, a three-element Maxwell rheological model combining a strain energy density function of high-damping rubber materials was proposed for modeling the cyclic tension and compression process. On the basis of the obtained results, an iterative identification procedure was used to identify the parameters involved in the constitutive model for each loading-unloading cycle. Using these parameters, the cyclic tension and compression of the material were predicted for different loading-unloading cycles. The identified constitutive parameters were further expressed as a function of the number of cycles. This provided an efficient way to investigate the impact of the Mullins and residual strain effects on the hyper-viscoelastic properties of the high-damping rubber material. Evolutions of hyperelastic parameters implied that the cyclic tension and compression of the material were mainly affected by the first stress invariant instead of the second stress invariant. In addition, evolutions of viscoelastic coefficients indicated that the elastic response of the material was enhanced while its viscous response was weakened during the process; namely, the high-damping rubber material hardened to some extent during the cyclic tension and compression process in the vertical direction. This could be further validated by the decreasing trend of relaxation times. In brief, these findings could probably explain the variations of the vertical stiffness and the damping energy dissipation of high-damping rubber isolation bearings during the cyclic loading-unloading process in practical engineering applications, which eventually results in the degradation of the isolation performance of the high-damping rubber isolation bearing in the vertical direction. Since the high-damping rubber material was assumed incompressible and isotropic in the constitutive model used in this work, a mismatch between the model prediction and the experimental data were observed for the first cycle during the cyclic tension process and all cycles during the cyclic compression process. Thus, considering the effect of the volumetric deformation and the anisotropic change in the material properties, further improvement on this constitutive model will be made in future works.

These investigations could facilitate the development of computational tools, which would regulate fundamental guidelines for the better control and optimization of the isolation performance of the high-damping rubber material used for high-damping rubber isolation bearings, which have a wider perspective of applications in the urban rail transit system. In addition, the creep or stress relaxation behavior of the high-damping rubber material will be produced under certain loading conditions, and the temperature rise in the material will be caused by hysteretic heat generation. These phenomena would also affect the mechanical and damping characteristics of high-damping rubber materials likewise. Thus, the effects of different loading rates and temperatures on the isolation performance of this high-damping rubber material should be investigated in the future.

## Figures and Tables

**Table 1:** Basic formulation of the rubber material (unit: parts per hundred rubbers [phr]).

Ingredient (phr)	Formulas	
	NR	NBR
Raw rubber	80–90	10–20
Carbon black (N330)	30–50	
ZnO	6–9	
Stearic acid	3–6	
S	1–4	
TMTD	0.1–1	
CZ	1–4	
4010NA	2–4	
Naphthenic oil	1–3	

ZnO—zinc oxide; S—sulfur; TMTD—Tetramethylthiuram disulfide; CZ—N-cyclohexylbenzothiazole-2-sulphenamide; 4010NA—N-isopropyl-N’-phenyl-p-phenylenediamine.

**Table 2:** Identified values of constitutive parameters during the cyclic tension and compression process.

Cyclic Tension Process					
Parameter	Number of Cycles				
	1	2	3	4	5
$c_{4}$	1.128	1.227	1.137	1.091	1.069
$c_{5}$	0.002	0.001	0.001	0.001	0.001
c	1.853	3.828	3.356	3.154	3.112
m	0.394	3.521	3.688	3.769	3.717
$g_{\infty }$	0.008	0.084	0.091	0.093	0.092
$g_{1}$	0.760	0.606	0.611	0.600	0.595
$g_{2}$	0.225	0.228	0.208	0.207	0.209
$g_{3}$	0.008	0.081	0.091	0.100	0.104
$\tau_{1}$	5.060	5.034	5.003	4.990	4.983
$\tau_{2}$	141.588	138.678	135.371	133.886	133.144
$\tau_{3}$	3961.939	3820.585	3662.524	3592.526	3557.718
Cyclic compression process					
Parameter	Number of cycles				
	1	2	3	4	5
$c_{4}$	5.877	6.139	5.547	5.367	5.650
$c_{5}$	0.002	0.002	0.002	0.002	0.002
c	1.898	1.096	0.990	0.951	0.908
m	5	4	4	4	4
$g_{\infty }$	0.158	0.180	0.201	0.207	0.201
$g_{1}$	0.669	0.680	0.641	0.633	0.650
$g_{2}$	0.171	0.139	0.156	0.158	0.148
$g_{3}$	0.001	0.002	0.002	0.002	0.001
$\tau_{1}$	4.410	4.224	4.189	4.178	4.158
$\tau_{2}$	81.667	68.762	66.497	65.790	64.545
$\tau_{3}$	1512.486	1119.366	1055.602	1036.054	1002.013

**Table 3:** Values of $R^{2}$ for each loading-unloading cycle during the cyclic tension and compression process.

Process	Number of Cycles				
	1	2	3	4	5
Tension	0.9561	0.9967	0.9978	0.9979	0.9978
Compression	0.9613	0.9780	0.9757	0.9764	0.9798

## Data Availability

Not applicable.
